# Exploring student thoughts and perception of videos as a learning resource

**DOI:** 10.1192/bjo.2021.381

**Published:** 2021-06-18

**Authors:** Gianluca Di Pasqua, Pranav Mahajan

**Affiliations:** University of Sheffield

## Abstract

**Aims:**

As medical education becomes increasingly digital, there is a plethora of readily available video resources available to medical students, aimed at teaching a wide range of topics. Despite this abundance, students report a myriad of issues. These range from videos containing outdated material, being of a poor production quality, and not being entirely relevant to their learning objectives. The aim of this study is to explore student thoughts and perceptions of videos as a teaching and learning resource. As the Mental State Examination is a component of the Psychiatry curriculum that students often find difficult, we have written, filmed and produced a video series explaining and demonstrating it.

**Method:**

Following the production of the Mental State Examination videos at the University of Sheffield – which contained multiple doctor-patient consultations, interspersed with narration outlining the key learning points – three focus groups were undertaken. These were aimed at understanding student thoughts and perception on the new videos, and the use of videos in medical education in general. Taking a qualitative approach, thematic analysis was performed on the content of the focus groups.

**Result:**

There was universal positive feedback about the structure and content of the videos we had produced; students enjoyed observing the various doctor-patient consultations and felt the separate elements of the Mental State Examination was explained logically and concisely. Furthermore, students appreciated that the videos were produced at their own University, believing this added to their validity. With regards to videos as a teaching resource in general, focus groups revealed that students appreciated specific, relevant and novel video material. Most students felt that videos can play an important role in medical education. There was a unanimous belief however that videos used in medical education should be produced well and be factually correct.

**Conclusion:**

Understanding how students feel about videos as a teaching and learning resource is crucial in the development of more in the future. This study suggests more video resources for many areas, both within Psychiatry, and within the greater sphere of medical education, would be welcomed. This ought to be accompanied with student evaluation.

